# Pharmacists in Primary Care: Catalyzing Treatment For Substance Use Disorders

**DOI:** 10.1007/s11606-025-09850-7

**Published:** 2025-09-22

**Authors:** Iraklis Erik Tseregounis, Clare C. Landefeld, Tonya L. Fancher

**Affiliations:** 1 Division of General Internal Medicine and Bioethics, Department of Internal Medicine, University of California, Davis School of Medicine, Sacramento, CA USA; 2 Division of General Internal Medicine and Primary Care, Department of Internal Medicine, Brigham and Women’s Hospital, Boston, MA USA

*“Does the SUD pharmacist have any open appointments today? I’m in with my patient who wants to talk about naltrexone.”*

Multiprofessional teams in busy primary care practices expedite and expand access to care for complex and life-threatening, while often ignored and stigmatizing, illnesses. During a routine primary care visit, a patient’s escalating alcohol use was brought up. In this instance, the multiprofessional substance use disorders (SUDs) team stepped in. Without delay, the SUDs team discussed medication options for unhealthy alcohol use while the primary care provider (PCP) used the rest of the visit to focus on other health and health maintenance priorities.

Low-barrier access to evidence-based SUD treatment is an enduring healthcare challenge.^1^ Primary care settings are crucial as they are often the only point of contact patients may have with healthcare providers. Challenges in primary care related to SUD pharmacotherapy—such as (historical) prescribing restrictions, variable experience in prescribing, workforce shortages, and stigma—continue to restrict timely access to life-saving treatment.^2^ Pharmacists are ideal partners to close this gap.

In this issue of JGIM, *Sterling et al. *^3^ examine the effectiveness of this coordinated care treatment approach to improving rates of pharmacotherapy and specialty addiction medicine treatment referrals among patients with heavy alcohol use or alcohol use disorder (AUD). This study utilized a cluster-randomized design to evaluate the effect of the Alcohol Telemedicine Consultation; an intervention that utilizes a telemedicine approach to connect pharmacists, trained to treat primary care patients with unhealthy alcohol use, with patients seen by PCPs. Sixteen adult primary care clinics within the Kaiser Permanente Northern California health system were randomized with half allocated to the intervention arm and the other half to usual care. Findings revealed that providers participating in the intervention had greater than 50% increases in medication order rates relative to clinics with usual care, and modest increases in order rates for specialty care referrals. While the generalizability of this study is somewhat limited to health systems with high levels of integration, such as Kaiser Permanente, findings indicate that pharmacist-based interventions may increase patient access to medications to treat SUD.

Identifying the best strategies for increasing treatment of SUD in primary care is timely. Given alcohol’s prevalence, stigma, and significant impacts on health, addressing heavy alcohol use and AUD is particularly important in primary care.^4^ However, with challenges navigating time constraints and competing priorities, PCPs face a tension between either seizing the opportunity to explore treatment options for a patient in the office or referring out to specialty care. A referral embedded in primary care is quick and convenient for patients, especially when considering the availability and effectiveness of alcohol pharmacotherapy,^5^ and low-barrier access to care is particularly important for treatment of SUD. One study found that rates of patient engagement in specialty addiction care were significantly higher for same- or next-day access compared to even 2 days out.^6^ In our clinical experience, “no-show” rates are still quite high for internal referrals to an embedded SUDs team. Future analysis of patient connection rates and associated treatment wait times in the *Sterling et al.* study would further clarify the benefits of the intervention approach.

Ultimately, as with other chronic conditions such as mood disorders and advanced heart failure, a spectrum of treatment options, including initial treatment by PCPs with guided escalation, will be most effective for treating AUD. Consider patients with depression, a commonly comorbid condition with AUD, which is routinely treated in primary care. If patients seeking care for depressive symptoms were consistently referred to specialists for medication treatment, as is often the case with AUD, then far fewer patients would receive treatment. As with depression treatment protocols, first-line treatment for AUD is ideally delivered in primary care. Enhancing PCP care with readily available pharmacists—and therefore minimizing burdens on PCPs while ensuring timely access to alcohol-related care—would help bridge existing treatment gaps for AUD pharmacotherapy.

As illustrated in this work by *Sterling et al.*, a telemedicine encounter by pharmacists with additional training in alcohol pharmacotherapy and with capacity to prescribe naltrexone can increase prescribing of alcohol pharmacotherapy. This work supports growing interest in telemedicine as an effective tool for reaching patients for evaluation and treatment of AUD.^7^ Other potential options include electronic consultations via the electronic medical record (EMR), EMR-clinical decision support tools as have been used for opioid use disorder,^8^ or the availability of clinic “champions,” PCPs with a focus on SUD who can serve as PCP peer support or even have dedicated visits to see clinic patients for SUD-related care.^9^

The findings from *Sterling et al.* should prompt policymakers, health systems, and training programs to rethink how to leverage every member of the care team to expand access to evidence-based treatments. More research is needed to examine the effectiveness and reach of this approach in less integrated health systems, to understand the effects of this approach on patient treatment retention and cessation outcomes, and in strategically developed incentives for further health system support. Furthermore, attention should be devoted to medical student and resident selection and training that meets the needs of communities and expand real-world collaborative clinical experiences.^10^ We fully support the potential for pharmacy colleagues to increase access to pharmacotherapy in primary care—a crucial and necessary step in managing withdrawal symptoms and improving chances for recovery.
